# Plasma Levels of microRNA-145 Are Associated with Severity of Coronary Artery Disease

**DOI:** 10.1371/journal.pone.0123477

**Published:** 2015-05-04

**Authors:** Hai Gao, Raviteja Reddy Guddeti, Yasushi Matsuzawa, Li-Ping Liu, Li-Xiao Su, Duo Guo, Shao-Ping Nie, Jie Du, Ming Zhang

**Affiliations:** 1 Department of Cardiology, Beijing Anzhen Hospital, Capital Medical University, Beijing, China; 2 Division of Cardiovascular Diseases, Mayo College of Medicine, Rochester, Minnesota, United States of America; 3 Beijing Institute of Heart, Lung and Blood Vessel Disease, Beijing, China; 4 Department of Nephrology, First Hospital of Tsinghua University, Beijing, China; 5 Department of Biostatistics, School of Public Health, Rutgers, The State University of New Jersey, Newark, United States of America; 6 Department of Histology and Embryology, School of Basic Medical Sciences, Capital Medical University, Beijing, China; Medical University Hamburg, University Heart Center, GERMANY

## Abstract

**Background and Objective:**

MicroRNAs (miRNAs) have been shown to be associated with various physiological and pathological conditions, including inflammation and cardiovascular disease, but little is known about their relationship with the presence of coronary artery disease (CAD) and disease severity.

**Methods:**

A total of 195 consecutive subjects who underwent coronary angiography for chest pain evaluation were enrolled in this study. In CAD patients severity of coronary lesions was assessed by the number of diseased vessels and the Synergy between PCI with Taxus and Cardiac surgery score (SYNTAX score). Plasma levels of miRNA-145 were quantified by real-time quantitative polymerase chain reaction test, and logarithmic transformation of miRNA-145 levels (Ln_miRNA-145) was used for analyses due to its skewed distribution.

**Results:**

Of the 195 total subjects 167 patients were diagnosed as having CAD. Ln_miRNA-145 was significantly lower in CAD patients compared with the non-CAD group (-6.11±0.92 vs. -5.06±1.25; *p* <0.001). In multivariable linear regression analyses CAD was significantly associated with lower Ln_miRNA-145 (Estimate, -0.50; standard error (SE), 0.11; *p* <0.0001). Furthermore, among CAD patients, three-vessel disease, higher SYNTAX scores and STEMI were significantly associated with lower Ln_miRNA-145 ([Estimate, -0.40; SE, 0.07; *p* <0.0001]; [Estimate, -0.02, SE, 0.10; *p* = 0.005] and [Estimate, -0.35, SE, 0.10; *p* <0.001] respectively).

**Conclusions:**

Lower plasma levels of miRNA-145 were significantly associated with the presence as well as severity of CAD. As a potential biomarker for CAD, plasma miRNA-145 may be useful in predicting CAD and its severity in patients presenting with chest pain.

## Introduction

Despite recent progress in the diagnosis and treatment of cardiovascular diseases, coronary artery disease (CAD) and its clinical residues still represent a major cause of morbidity and mortality worldwide [1–3]. Atherosclerosis, which is the major cause of CAD, is now widely considered as a chronic inflammatory state [4]. Vascular smooth muscle cells (VSMCs) play a critical role in the pathogenesis of a variety of cardiovascular diseases such as atherosclerosis, aneurysms and re-stenosis after angioplasty or bypass [5,6]. Conversion of VSMC phenotype from differentiated to dedifferentiated is known to be associated with marked increase in VSMC expression of inflammatory cytokines which facilitate monocyte and macrophage adhesion promoting plaque formation [7]. Recent studies have demonstrated that micro ribonucleic acids (miRNAs), which are small, single-stranded, non-coding RNA molecules known to be essential as post-transcriptional modulators of gene expression and coordinators of several regulatory pathways involved in cell differentiation and proliferation, are associated with various physiological and pathological conditions, including inflammation and cardiovascular disease [8–11]. The proposed mechanisms by which miRNAs regulate cellular differentiation and proliferation include either translational repression or RNA degradation [8]. MiRNAs are predicted to control and regulate approximately 60% of all coding genes. Plasma miRNAs have been considered as promising novel biomarkers for the diagnosis and prognosis of cardiovascular diseases, especially CAD [12,13].

Several lines of evidence suggest that miRNA-145 is involved in the regulation of VSMC proliferation, differentiation and phenotype [5]. MiRNA-145 is also one of the most abundant miRNA in normal, healthy arterial walls, and VSMCs and has been shown to be the major determinant of VSMC phenotype in vivo. Experimental animal models have reported that expression of miRNA-145 in arteries is attenuated in vascular injury, atherosclerosis and aneurysms [14,15]. Although expression of different circulating miRNAs in CAD patients has been reported [16], the role of miRNA-145 in atherosclerosis has not been studied extensively among different presentations of CAD.

In the present study, we aimed to determine the association between plasma miRNA-145 levels and the presence and severity of CAD.

## Methods

### Study subjects

Between 5/28/2012 and 12/17/2013 a total of 195 consecutive patients who underwent diagnostic coronary angiography for chest pain evaluation were recruited from inpatients admitted to Beijing Anzhen Hospital of Capital Medical University. Coronary angiography was performed via transfemoral or transradial approach using techniques, equipment and practices standard to Beijing Anzhen Hospital’s cardiac catheterization laboratory. Diagnosis of CAD was confirmed by coronary angiography and was defined as angiographic evidence of more than 50% luminal narrowing in at least one segment of a main epicardial coronary artery [17]. CAD patients were again categorized into ST-elevation myocardial infarction (STEMI), unstable angina/non-ST-elevation MI (UA/NSTEMI) and stable angina based on standard ACC/AHA definitions. STEMI and UA/NSTEMI patients constituted ACS group. Subjects with no angiographic evidence of CAD were grouped under non-CAD.

Patients with histories of significant concomitant diseases including hepatic failure, renal failure, hepatitis, cardiomyopathy, congenital heart disease, bleeding disorders, previous thoracic irradiation therapy, and malignant diseases were excluded from the study.

### Ethics statement

The study protocol was approved by the Beijing Anzhen Hospital Ethics Committee of Capital Medical University, and a written informed consent was obtained from all the participants.

### Plasma collection and storage

Blood samples were collected in EDTA-containing tubes (BD, Franklin Lakes, NJ, USA) and plasma was isolated within 1 hour by centrifugation at 1900×g for 10 minutes at 4°C to remove blood cells, and then at 16,000×g for 10 minutes at 4°C to remove additional cellular nucleic acids attached to cell debris, plasma samples were transferred to RNase/Dnase-free tubes and stored at -80°C. Studies have shown that heparin administration as part of ACS treatment is associated with interference in plasma miRNA measurement [18]. Therefore in our study blood samples were collected before heparin boluses were administered, especially in patients presenting with unstable CAD features. In patients who presented with stable CAD samples were collected by the next day.

### RNA isolation and preparation

Total RNA in plasma was isolated by the use of miRNeasy Plasma Kit (QIAGEN, Hilden, Germany) following the manufacturer's instructions. In detail, 200 μL EDTA-containing plasma was mixed thoroughly with 1000 μL QIAzol in RNase-free tube, incubated for 5 minutes at room temperature (15~25°C). To normalize for the plasma miRNA content, we supplemented 3.5 μL (1.6 × 10^8^ copies/μL) *Caenorhabditis elegans* miR-39 (cel-miR-39) to the samples (after addition of QIAzol) [19]. After mixing thoroughly, 200 μL of chloroform was added into the mix. Following vigorous vortex mix for 15 seconds, the tube containing the lysate was placed at room temperature for 2~3 minutes and then centrifuged for 15 minutes at 12000×g at 4°C. Later, the aqueous phase containing the RNA was carefully removed.

### Quantification of microRNA-145

Total RNA was poly-adenylated and reverse transcribed using the miScript Reverse Transcription kit (QIAGEN) as per the manufacturer's instructions with 100~200 ng of total RNA per reaction. The obtained cDNA (2.5 μL of a 1/100 dilution) was then PCR-amplified using the miScript SYBR Green PCR kit (QIAGEN) in 25 μL reaction mixtures consisting of 12.5 μL Quantitect SYBR Green PCR master mix and 2.5ul miR-145 primer and the miScript universal primer. The reactions were incubated at 95°C for 15 minutes, followed by 45 cycles of 94°C for 15 seconds, 55°C for 30 seconds, and 70°C for 30 seconds. The relative expression level for miR-145 was computed using the comparative Ct method. MiRNA expression was normalized to cel-miR-39 and expressed as 2^− (CT [microRNA] −CT [cel-miR-39])^ [19]

### Assessment of CAD severity

Depending on the extent of epicardial coronary artery involvement CAD patients were divided into single-, double-, and triple-vessel disease subgroups in accordance with the Coronary Artery Surgery Study classification [20,21]. Severity of coronary lesions was assessed using the Synergy between PCI with Taxus and Cardiac surgery score (SYNTAX score), an angiographic score to determine the severity and complexity of the disease [22]. Total SYNTAX score for each patient was calculated by summing the total points assigned to each individual lesion in coronary arteries >50% stenosis and >1.5 mm diameter. Two cardiologists, who were blinded to the study patient data, assessed the angiograms and SYNTAX scores were measured accordingly.

### Statistical analysis

Normality of distribution was assessed using Kolmogorov-Smirnov test. In our study the distribution of plasma miRNA-145 values among the study subjects was skewed and therefore logarithmic transformation was performed to normalize plasma miRNA-145 data (Ln_miRNA-145). Comparisons between 2 groups were performed using Fisher’s test, Student’s t test or Mann–Whitney U test. For comparison of more than 2 groups, one-way ANOVA and Tukey-Kramer HSD (honestly significant difference) tests were used as appropriate. Pearson χ^2^ test and Spearman ρ test were used to compare qualitative and quantitative variables as appropriate. Correlations between plasma levels of miRNAs and other variables were calculated using Pearson correlation coefficient for symmetric distributions and Spearman’s coefficient for skewed variables. Univariate and multivariable linear regression analyses were performed to determine variables that were associated with lower Ln_miRNA-145. All tests were two-sided and a *p* <0.05 was considered statistically significant. All statistical analyses were performed using JMP 10 software (SAS Institute, Inc., Cary, NC, USA).

## Results

### Basic clinical characteristics of patients

Table 1 demonstrates basic clinical characteristics in the whole population. Mean age was 59.5±11 years and males constituted 70% of the total subjects. Prevalence of traditional CAD risk factors, such as hypertension, diabetes mellitus, dyslipidemia and smoking was 70%, 37%, 37% and 45% respectively (Table 1 and S1 Table). Mean Ln_miRNA-145 in the whole population was -6.0±1.0. Of the 195 patients 167 were diagnosed as having CAD and the rest of them were grouped under non-CAD (n = 28).

**Table 1:** Basic patient characteristics in the whole population.

Basic clinical characteristics	Whole population (n = 195)
**Age, yr**	59.5±11
**Male Gender, n (%)**	136 (70)
**Body mass index, kg/m** ^**2**^	26±3.6
**Hypertension, n (%)**	137 (70)
**Diabetes, n (%)**	73 (37)
**Dyslipidemia, n (%)**	72 (37)
**Smoking, n (%)**	87 (45)
**Blood glucose, mmol/L**	5.5 (5.1, 6.5)
**Triglycerides, mmol/L**	1.4 (1.0, 2.2)
**Total cholesterol, mmol/L**	4.2±1.0
**Low-density lipoprotein, mmol/L**	2.6±0.9
**High-density lipoprotein, mmol/L**	1.0±0.2
**hsCRP, mg/L**	1.4 (0.7, 4.9)
**Serum creatinine, μmol/L**	73±15
**Number of diseased vessels, n (%)**	0 = 28 (14.3), 1 = 34 (17.3), 2 = 60 (31), 3 = 73 (37.4)
**Ejection fraction, %**	61±8
**LVEDD, mm**	49.4±5.5
**Ln_miRNA-145**	-6.0±1.0

Continuous data are expressed as mean ± standard deviation or medial with interquartile range; Categorical data are expressed as frequencies; CAD, Coronary artery disease; hsCRP, High sensitivity C-reactive protein; Ln, Logarithmic; LVEDD, Left ventricular end diastolic diameter.

### Association of plasma miRNA-145 levels and traditional risk factors for CAD

Table 2 demonstrates univariate linear regression analysis for the association of clinical variables and Ln_miRNA-145. Diabetes and smoking were significantly (*p* = 0.032 and 0.008 respectively) and male gender was non-significantly (*p* = 0.070) associated with lower Ln_miRNA-145 (Table 2). Although dyslipidemia showed a tendency for negative association with miRNA-145 levels, the *p* value was not statistically significant (*p* = 0.12).

**Table 2:** Univariate linear regression analysis for plasma levels of Ln_miRNA-145.

Variable	Estimate	Standard error	*p*
**Age**	0.004	0.007	0.52
**Male gender**	-0.15	0.08	0.070
**Body mass index**	-0.01	0.02	0.59
**Diabetes mellitus**	-0.16	0.08	0.032*
**Hypertension**	-0.04	0.08	0.64
**Dyslipidemia**	-0.12	0.08	0.12
**Smoking**	-0.22	0.07	0.002*

* *p*<0.05; Data are expressed as parameter estimates with standard errors. All continuous variables were standardized to a mean of 0 and a standard deviation of 1; all dichotomous variables were coded as 0 = presence and 1 = absence; CAD, Coronary artery disease.

### Reduced plasma miRNA-145 levels are associated with the presence of CAD

CAD patients had significantly lower plasma levels of miRNA-145 compared with non-CAD subjects (Ln_miRNA-145 -6.11±0.92 vs. -5.06±1.25; *p* <0.001). CAD was associated with lower Ln_miRNA-145 both in univariate linear regression analysis (Estimate, -0.52; standard error, 0.1; *p* <0.0001) and multivariate linear regression analysis (Estimate, -0.50; standard error, 0.11; *p* <0.0001) after adjustment for traditional coronary risk factors (Table 3). Interestingly, in the current study plasma miRNA-145 levels significantly correlated with higher ejection fraction (Spearman ρ = 0.18; *p* = 0.014) and larger left ventricular end-diastolic diameter (Spearman ρ = -0.26; *p* < 0.001) respectively.

**Table 3:** Association of miRNA-145 with severity of CAD.

	Univariate			Adjusted for traditional risk factors		
	Estimate	SE	*P*	Estimate	SE	*P*
**In whole population (n = 195)**						
CAD	-0.52	0.1	<0.0001*	-0.50	0.11	<0.0001*
**In CAD patients (n = 167)**						
Number of diseased vessel	-0.40	0.06	<0.0001*	-0.40	0.07	<0.0001*
SYNTAX score	-0.02	0.006	0.003*	-0.02	0.007	0.005*
STEMI	-0.41	0.09	<0.0001*	-0.35	0.10	<0.001*

* *p*<0.05; CAD, Coronary artery disease; SE, Standard error; STEMI, ST-elevation myocardial infarction; Data are expressed as parameter estimates with standard errors. All continuous variables were standardized to a mean of 0 and a standard deviation of 1; all dichotomous variables were coded as 0 = presence and 1 = absence. Traditional risk factors include age, male gender, body mass index, diabetes, hypertension, dyslipidemia, smoking. SYNTAX groups were represented as continuous values.

### Association of plasma miRNA-145 levels with severity of CAD

**Fig 1:**
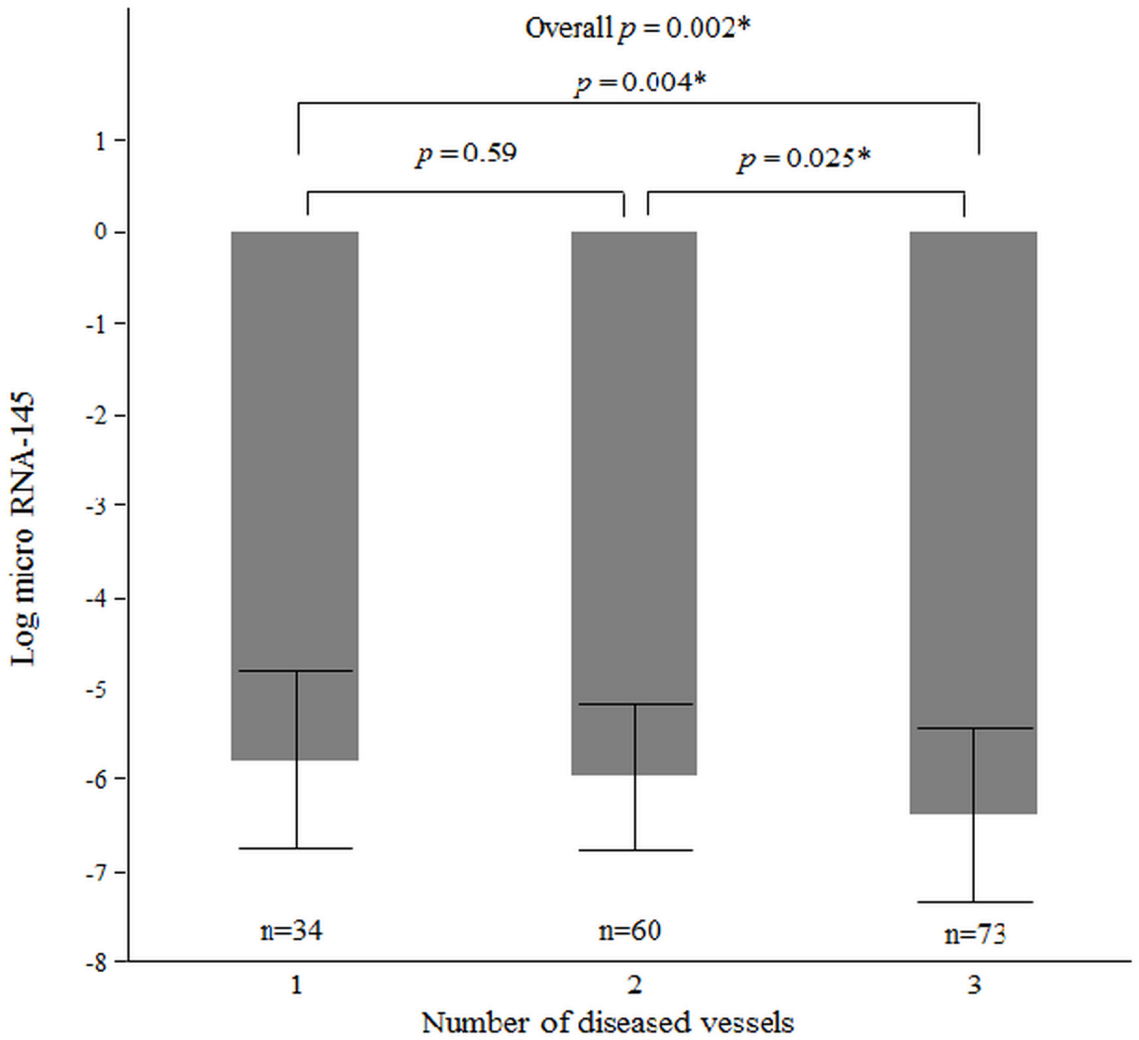
Association between plasma miRNA-145 levels and number of diseased vessels. These bars represent averages of plasma miRNA-145 levels among patients with single-, double- and triple-vessel CAD. T-bars indicate standard deviation. Patients with three-vessel disease have significantly lower levels of miRNA-145 compared with the rest of the groups. Levels were not significantly different between patients with single- and double-vessel CAD. * *p* <0.05; CAD, coronary artery disease, RNA, Ribonucleic acid.

To further evaluate the relationship between plasma miRNA-145 levels and CAD we performed a sub-analysis in CAD population (n = 167) to determine the association between plasma levels of miRNA-145 and severity of CAD. Severity of CAD was assessed by number of diseased vessels, SYNTAX score and the type of CAD at presentation. By coronary angiography, 34 (20%) CAD patients had single-vessel disease, 60 (36%) had double-vessel disease and 73 (44%) had triple-vessel disease. The levels of miRNA-145 in patients with three-vessel disease were significantly lower than those with one or two-vessel disease (Ln_miRNA-145 -6.38±0.94 vs. -5.78±0.98; *p* = 0.002 and -6.38±0.94 vs. -5.98±0.79; *p* = 0.025, respectively) (“Fig 1”). However, our study results showed that the levels between patients with one-vessel and two-vessel disease were not significantly different (p = 0.59) (“Fig 1”). In univariate (Estimate, -0.40, standard error, 0.06; *p* <0.0001) and multivariable linear regression (Estimate, -0.40, standard error, 0.07; *p* <0.0001) analyses higher number of diseased vessels was significantly associated with lower Ln_miRNA-145 (Table 3).

**Fig 2:**
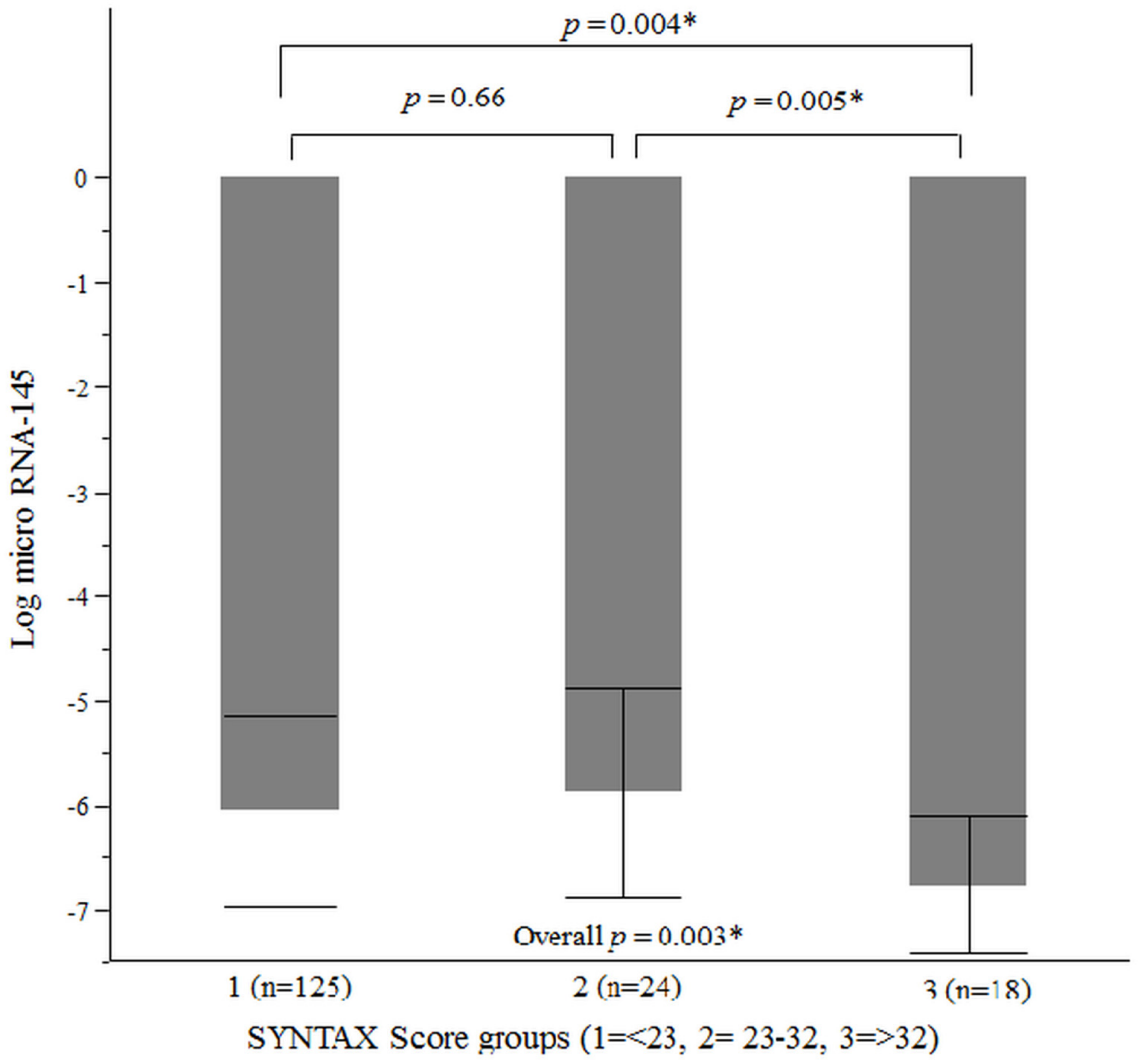
SYNTAX groups and plasma miRNA-145. These bars represent averages of plasma miRNA-145 levels across SYNTAX groups. T-bars indicate standard deviation. It can be noted that patients with high risk SYNTAX scores had significantly lower Ln_miRNA-145 compared with low and moderate risk groups. * *p* <0.05; RNA, Ribonucleic acid; SYNTAX, Synergy Between PCI With Taxus and Cardiac Surgery.

**Fig 3:**
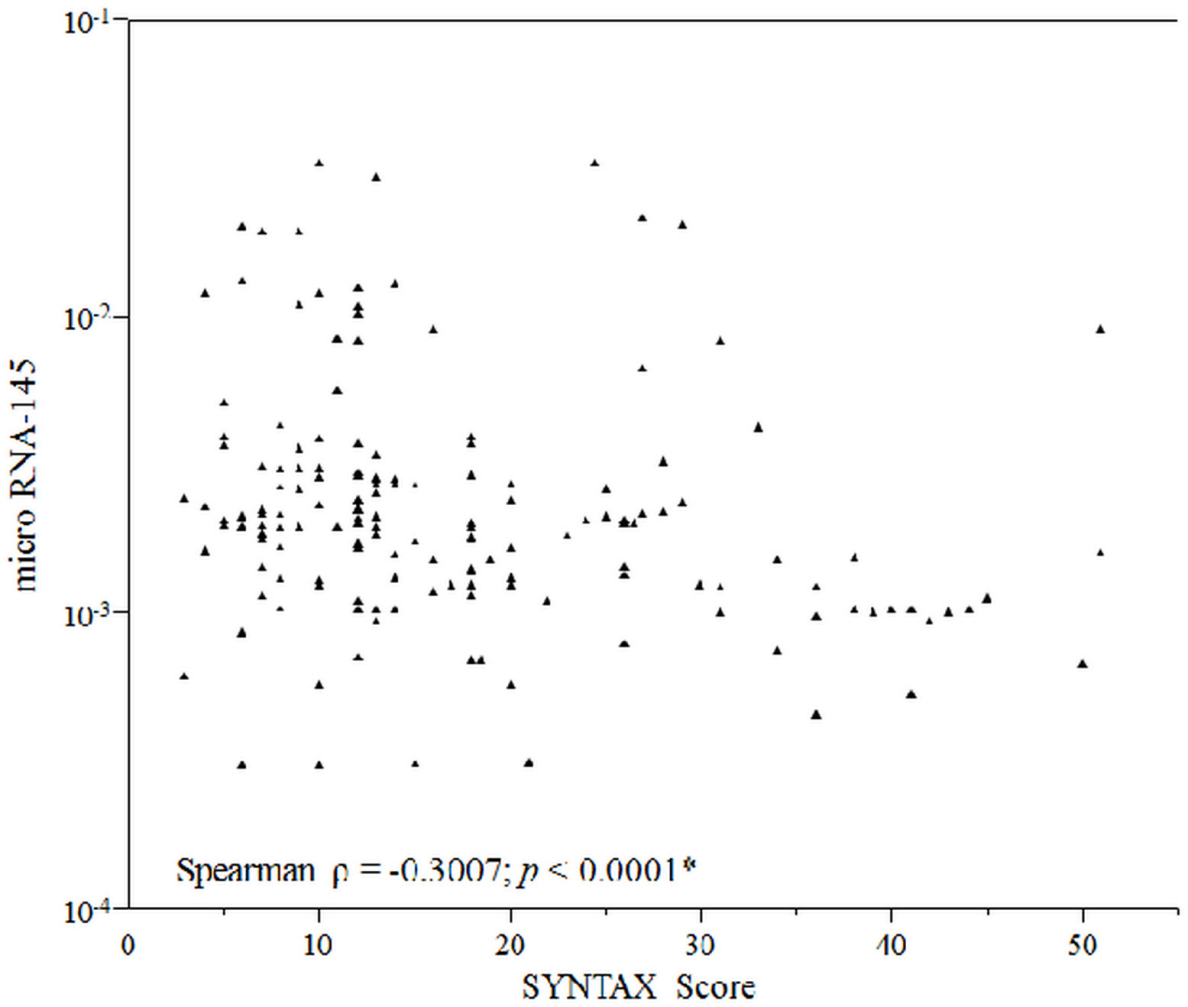
Correlation between SYNTAX score and plasma miRNA-145. The above scatter diagram demonstrates correlation between miRNA-145 and SYNTAX score. It can be noted that increase in SYNTAX score (increase in severity and complexity of CAD) is significantly correlated with reduced plasma miRNA-145 levels. * *p* <0.05; CAD, Coronary artery disease; RNA, Ribonucleic acid; SYNTAX, Synergy between PCI With Taxus and Cardiac Surgery.

On the basis of their SYNTAX scores CAD patients were again divided into three groups; low (SYNTAX score <23; n = 125), intermediate (SYNTAX score 23–32; n = 24) and high (SYNTAX score >32; n = 18) and the plasma miRNA-145 levels in each group were assessed. Patients with high SYNTAX score had significantly lower Ln_miRNA-145 levels compared with those with low and intermediate score (Low score group -6.05±0.91, Intermediate score group -5.88±1.00, High score group -6.76±0.66; *p* = 0.004 [low vs. high score groups] and *p* = 0.005 [intermediate vs. high score groups], respectively) (“Fig 2”). Although our study data demonstrated significant differences in miRNA-145 levels between low SYNTAX score versus high score, and intermediate score versus high score groups, no significant differences were observed between low score and intermediate score groups (*p* = 0.66). Lower plasma levels of miRNA-145 significantly correlated with higher SYNTAX scores (Spearman ρ = -0.30; *p* <0.0001), indicating reduced plasma miRNA-145 levels with increase in severity and complexity of CAD (“Fig 3”). In univariate and multivariable linear regression analyses higher SYNTAX score was significantly associated with lower Ln_miRNA-145 (Estimate, -0.02; standard error, 0.006; *p* = 0.003 and Estimate, -0.02; standard error, 0.007; *p* = 0.005, respectively) (Table 3).

### Altered expression of miRNA-145 in patients with ST-elevation myocardial infarction

**Fig 4:**
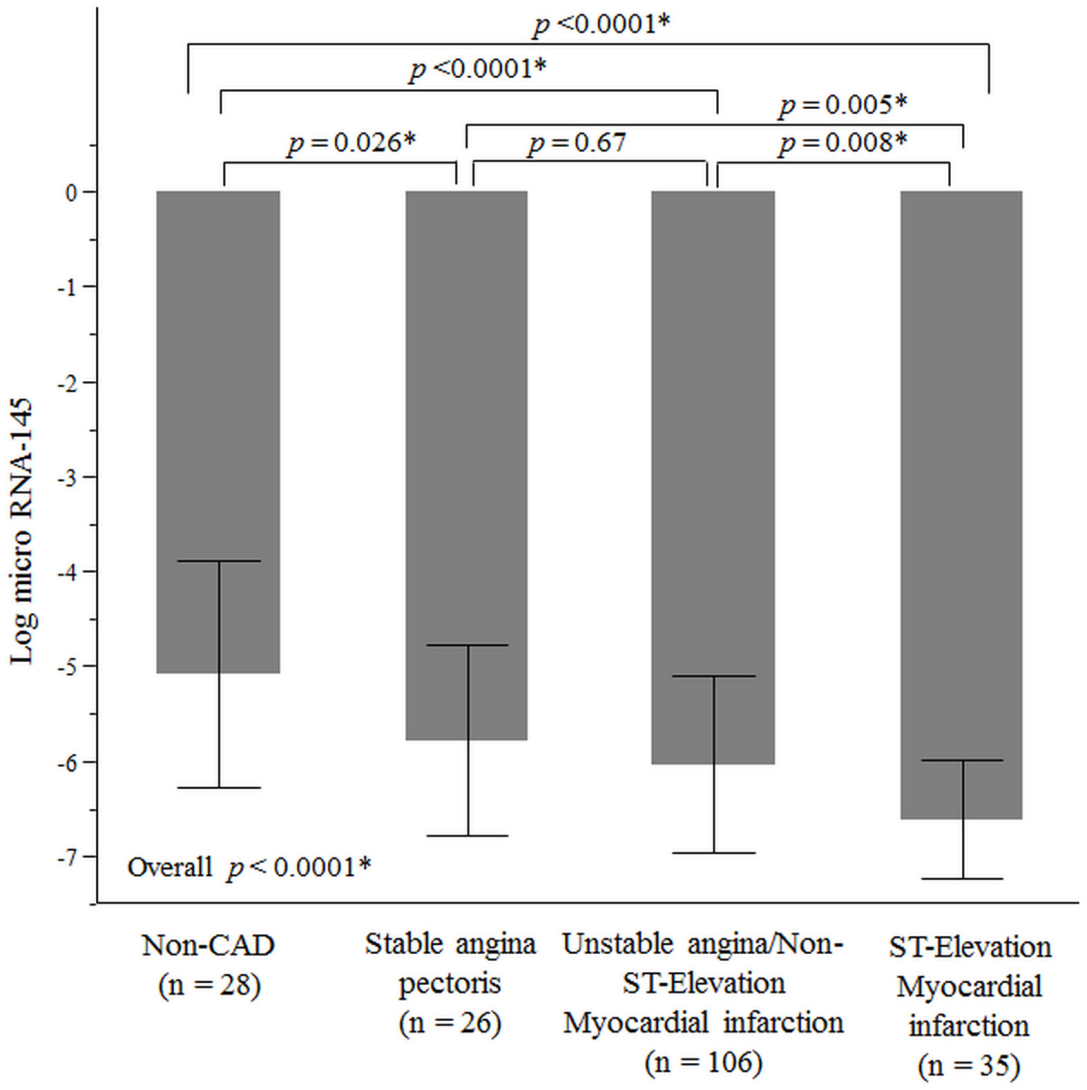
Plasma miRNA-145 levels and clinical presentation of CAD. These bars represent averages of Ln_miRNA-145 values among different clinical presentations of CAD compared with non-CAD patients. T-bars indicate standard deviation. Patients with ST-elevation myocardial infarction had significantly lower values of Ln_miRNA-145 compared with the rest of the groups. Non-CAD subjects had significantly higher Ln_miRNA-145 than patients with CAD. * *p* <0.05; CAD, Coronary artery disease; RNA, Ribonucleic acid.

Levels of plasma miRNA-145 were lower in patients with ACS, especially STEMI. “Fig 4” demonstrates the relative differences in Ln_miRNA-145 values between non-CAD (n = 28), stable angina pectoris (n = 26), UA/NSTEMI (n = 106) and STEMI (n = 35). It can be noted that STEMI patients had significantly lower Ln_miRNA-145 compared with the other groups indicating an altered expression of miRNA-145 in these patients. STEMI was associated with significantly lower Ln_miRNA-145 in multivariable linear regression analysis after adjustment for traditional CAD risk factors (Estimate, -0.35; standard error, 0.10; *p* <0.001 respectively) (Table 3). However, in our study miRNA-145 levels between stable angina and UA/NSTEMI were not found to be significantly different (*p* = 0.67).

## Discussion

In summary, our study findings demonstrate that plasma levels of miRNA-145 are significantly lower in CAD patients compared with non-CAD subjects. These results reiterate findings from previous study that demonstrated significantly lower miRNA-145 levels in CAD patients [19]. Also, in our study we demonstrate that with increase in severity of CAD (multi-vessel disease and higher SYNTAX score) the levels are reduced further more. Within the CAD group, patients presenting with ACS, especially STEMI, had significantly lower miRNA-145 levels compared to other groups. However, it should also be noted that plasma miRNA-145 levels were not significantly different among stable angina and UA/NSTEMI patients.

### Roles of miRNA-145 in atherosclerosis

VSMCs are thought to play a significant role in the pathogenesis of atherosclerosis. They are known to possess remarkable plasticity that allows them to reversibly modulate their phenotype during endothelial injury, inflammation and vessel wall injury [23]. Phenotype switching from quiescent ‘contractile’ or differentiated type to active ‘synthetic’ or dedifferentiated form is associated with enhanced proliferative rate, migration and formation of fibrous plaque [15,24]. In addition to the above ‘synthetic’ phenotype VSMCs also facilitate pathological lipid uptake and foam cell formation by enhanced expression of low-density lipoprotein, very low-density lipoprotein and scavenger receptors [23]. Circulating miRNA-145 has been shown to be regulated during coronary atherosclerosis [14,25]. Cheng Y et al [15] reported that miRNA-145 is the most abundant miRNA subtype in the normal vascular walls and that it is selectively expressed in the VSMCs of vessel wall, and can regulate the phenotype of VSMC. Experimental animal studies have reported that carotid artery ligation was associated with markedly reduced miRNA-145 levels, as measured by quantitative PCR and subsequent carotid stenosis [14]. Cordes and colleagues demonstrated that miRNA-145 promotes differentiation and represses proliferation of SMCs and the levels of this miRNA subtype were downregulated to nearly undetectable levels in atherosclerotic lesions containing neointimal hyperplasia [14]. Mechanisms by which miRNA-145 regulates VSMC phenotype include KLF4, calmodulin kinase IIδ, myocardin, actin polymerization and angiotensin-converting enzyme [26]. Restoration of miRNA-145 expression limits neointima formation in response to vascular injury by promoting KLF4 down regulation and VSMC contractile protein expression [15,26].

### Circulating miRNA-145 as potential biomarkers for CAD

In this study, compared with that of controls, we demonstrated that plasma levels of miRNA-145 were significantly decreased in patients with CAD. These results suggest that downregulation of miRNA-145 plays a critical role in the pathogenesis of atherosclerotic plaques. Also, in the present study, miRNA-145 was correlated with multiple metabolic and CAD risk factors, including age, gender, diabetes mellitus, and tobacco use. Our study findings also show that miRNA-145 levels are associated negatively with the severity and extent of coronary stenotic lesions indicating further reduction in its plasma levels with increase in severity of CAD. CAD patients with multivessel disease, higher SYNTAX score and those with STEMI were associated with significantly lower miRNA-145 levels compared with others. To the best of our knowledge this is the first study to report on the association between plasma miRNA-145 levels and severity of CAD, especially patients with STEMI and high SYNTAX score, which further suggest that miRNA-145 might serve as biomarkers of CAD development and progression.

Several studies have in the past demonstrated the important of circulating microRNAs as potential biomarkers for CAD [27–29]. VSMCs play an important role in the stability and plaque rupture mechanisms [30]. While miRNA-145 can regulate vascular smooth muscle cell phenotype and extracellular matrix synthesis, thus possibly affecting the stability of the plaque, this article discusses the potential of plasma miRNA-145 as a clinical biomarker for the diagnosis and prognosis of acute coronary syndromes. However, it should be noted that using PCR to measure plasma miRNAs is expensive and time consuming, and there is a definite need to develop semi-automated techniques in the coming future to overcome this limitation.

### MiRNA-145 as a potential therapeutic target for atherosclerosis

With the ability to regulate phenotypic differentiation of VSMCs, miRNA-145 can also act as a potential therapeutic target in the treatment of atherosclerosis [31,32]. Restoration of downregulated plasma miRNA-145 might inhibit neointimal lesion formation in the coronary arteries [14,32]. In a recent study by Lovren et al in experimental atherosclerosis it was demonstrated that targeted delivery of miRNA-145 into VSMCs in Apolipoprotein E knockout mice (*ApoE* ^*-/-*^) was associated with markedly reduced plaque size, significantly increased fibrous cap area and plaque collagen content, and reduced necrotic core area at 12 weeks [32]. The study noted that apart from reducing overall atheroma burden, lentivirus mediated miRNA-145 transfer was associated with increased plaque stability thereby concluding that VSMC-specific miRNA-145 overexpression could be a novel therapeutic target in the management of atherosclerosis. However, further studies are warranted to establish miRNA-145 as a therapeutic option in atherosclerosis.

### Limitations

Our study has several limitations. First, it is a single-center study involving a small sample size and therefore the results of the study should be interpreted with caution. Second, in our study we could not demonstrate the mechanisms behind the association between lower miRNA-145 levels in the plasma and CAD severity. Third, the distribution of patients in CAD and non-CAD groups was uneven. In our study we failed to demonstrate significant differences in miRNA-145 levels between patients with stable angina and unstable angina/non-ST-elevation myocardial infarction, and in patients with low and intermediate SYNTAX scores. Therefore our study results cannot be generalized to the whole CAD population. Large-scale multicenter studies are required to further elucidate the role of miRNA-145 as a potential marker in CAD, especially ACS. Finally, in our study we used *C*. *elegans* miRNA-39 as reference for normalization of miRNA-145 data. Some recent studies have reported using other housekeeping miRNAs for this purpose that might result in a different normalization data which might influence the overall results [33].

## Conclusion

In conclusion, plasma miRNA-145 levels are significantly lower in CAD patients compared with those without significant CAD and this reduction seemed to positively correlate with the severity of CAD. These findings may have extensive clinical implications in the diagnosis and therapy of CAD.

## Supporting Information

S1 Table Basic patient characteristics in the whole cohort. (DOCX)
