# Correction: Plasma Levels of microRNA-145 Are Associated with Severity of Coronary Artery Disease

**DOI:** 10.1371/journal.pone.0130780

**Published:** 2015-06-11

**Authors:** The PLOS ONE Staff

The eighth author and the ninth author, Jie Du and Ming Zhang, should both be noted as corresponding authors of this work. Jie Du can be contacted at jiedubj@126.com. The publisher apologizes for this error.
