# Supplementary material for: Plasma Levels of microRNA-145 Are Associated with Severity of Coronary Artery Disease
Source: PLoS One. 2015 May 4;10(5):e0123477. doi: 10.1371/journal.pone.0123477 (PMC4418743; doi:10.1371/journal.pone.0123477)
Supplement: S1 Table — (DOCX) [file pone.0123477.s001.docx]

**Supplemental Table 1:** Basic patient characteristics in the whole cohort

| **Basic clinical characteristics** | **Non-CAD (n=28)** | **CAD (n=167)** | ***p*** |
| --- | --- | --- | --- |
| **Age, yr** | 56±12 | 60±11 | 0.074 |
| **Male Gender, n (%)** | 11 (39) | 125 (75) | <0.001* |
| **Body mass index, kg/m^2^** | 26.7±3.8 | 26.1±3.5 | 0.48 |
| **Hypertension, n (%)** | 13 (46) | 124 (74) | 0.006* |
| **Diabetes, n (%)** | 5 (18) | 68 (41) | 0.021* |
| **Dyslipidemia, n (%)** | 6 (21) | 66 (40) | 0.090 |
| **Smoking, n (%)** | 7 (25) | 80 (48) | 0.025* |
| **Blood glucose, mmol/L** | 5.2 (5.0, 5.6) | 5.6 (5.1, 6.5) | 0.030* |
| **Triglycerides, mmol/L** | 1.6±0.8 | 1.8±1.3 | 0.33 |
| **Total cholesterol, mmol/L** | 4.7±0.9 | 4.1±1.1 | 0.001* |
| **Low-density lipoprotein, mmol/L** | 2.9±0.8 | 2.5±0.9 | 0.028* |
| **High-density lipoprotein, mmol/L** | 1.2±0.3 | 0.9±0.2 | <0.001* |
| **hsCRP, mg/L** | 1.2 (0.7, 2.2) | 1.7 (0.7, 5.9) | 0.13 |
| **Serum creatinine, µmol/L** | 67±12 | 74±15 | 0.004* |
| **Ejection fraction, %** | 66±8 | 61±8 | 0.003* |
| **LVEDD, mm** | 47±5 | 50±5 | 0.009* |
| **Ln_miRNA-145** | -5.06±1.25 | -6.11±0.92 | <0.001* |

* *p*<0.05; Continuous data are expressed as mean ± standard deviation or medial with interquartile range; Categorical data are expressed as frequencies; CAD, Coronary artery disease; hsCRP, High sensitivity C-reactive protein; Ln, Logarithmic; LVEDD, Left ventricular end diastolic diameter
